# A global perspective on the governance-health nexus

**DOI:** 10.1186/s12913-023-10261-9

**Published:** 2023-11-10

**Authors:** Mohammad Naim Azimi, Mohammad Mafizur Rahman, Son Nghiem

**Affiliations:** 1 School of Business, University of Southern Queensland, 487-535 West St, Toowoomba, Darling Heights, QLD 4350 Australia; 2 College of Health and Medicine, Australian National University, Canberra, ACT 2601 Australia

**Keywords:** Healthcare, Governance, Health expenditures, Income level, Cross-sectional dependence

## Abstract

**Background:**

This study raises two key arguments: First, government health expenditure (GHE) and per capita out-of-pocket expenditures on healthcare (OPEH) are sensitive to contemporary good governance practices, giving policy importance to the exogeneity of healthcare determinants, i.e., governance for health rather than health governance. Second, it is the income level of countries that reflects the volatility of the governance spillovers on the subject.

**Methods:**

The present study constructs a composite governance index (CGI) and employs a set of panel data for 144 countries over the period from 2002 to 2020. To allow comparability and extract specific policy implications, the countries are classified as full, high-, middle-, and low-income panels. Meanwhile to﻿ delve into the short- and long-run effects of CGI on GHE and OPEH, the study employs the cross-sectionally augmented autoregressive distributed lags (CS-ARDL) model. Further, to establish a causal link between the variables, it uses the Dumitrescu-Hurlin panel causality technique.

**Results:**

The results indicate that CGI is significantly cointegrated with GHE and OPEH in all recipient panels. It indicates that while CGI has significantly positive impacts on GHE and OPEH, its effects vary according to the income level of the underlying economies. The findings support the idea of governance for health and show that CGI drives the stabilization and enhancement of GHE and OPEH in the long run. Furthermore, the findings reveal that economic growth, the age dependency ratio, and tax revenue have positive effects, while the crude death rate and the child mortality rate exert negative impacts on the subject. Finally, the results highlight a unidirectional causality running from CGI to GHE and OPEH, while no feedback response is evident.

**Conclusions:**

Although an increase in GHE and OPEH is associated with the improvement of the population’s healthcare, the results suggest the recognition of the importance and institutionalization of good governance to streamline this improvement through effective channelization, outreach, and social environment development for extensive health inclusion.

**Supplementary Information:**

The online version contains supplementary material available at 10.1186/s12913-023-10261-9.

## Introduction

**Fig. 1:**
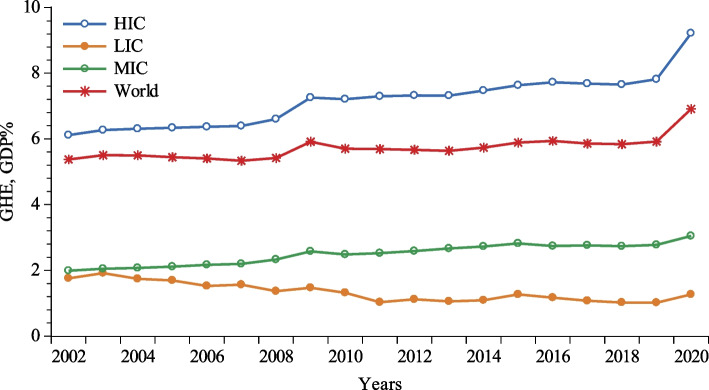
Government health expenditure (GHE, GDP%) plot.  Source: World Development Indicators (WDI) [6]

The ever-increasing discussion in contemporary literature about the effects of good governance on various macro- and socio-economic indicators and, as such, human capital well-being as a major driver of social and growth inclusion owes to the seminal work of North [1], which was further expanded by Lott and North [2] in the early 1990s. The contributions made by good governance in the purview of education, defense, justice, economic development, and human capital accumulation to offset potential market failures and promote economic output at macro-levels are undeniable [3]. However, establishing a link between good governance and health outcome indicators has often been controversial and a priori indeterminate, despite the fact that it has recently gained importance due to its emerging policy implications [4]. Moreover, it is well-evident that human well-being does not necessarily flourish in economies with sufficient resources; rather, it thrives when available resources are effectively accumulated and distributed through a well-designed bureaucratic system. Nevertheless, both government and out-of-pocket expenditures on healthcare have increased during the past two decades, while the percentage of mortality rates due to various health risk factors has also increased in parallel worldwide [5]. Figure 1 shows that government health expenditures have increased from 6.11% to 2002 to 9.21% of the GDP in 2020 in high-income economies, which is above the world’s average (6.9% in 2020). On the other hand, comparatively, while government health expenditures moved slightly higher in middle-income countries, catastrophically, they declined in low-income economies from 1.99% of the GDP in 2002 to 1.27% in 2020.

**Fig. 2:**
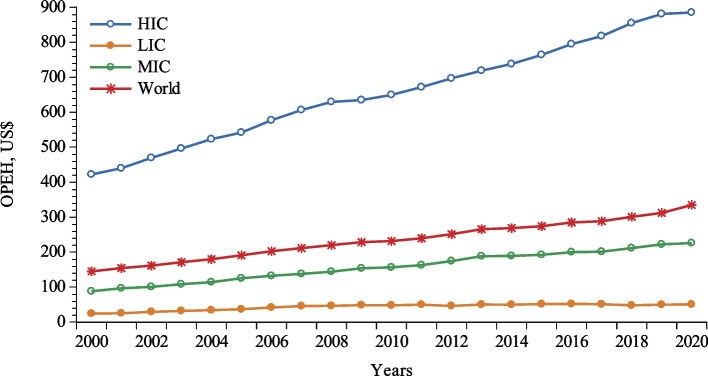
Per capita out-of-pocket expenditures on healthcare (OPEH) plot.  Source: World Health Organization (WHO) [17]

Almost a similar scenario can be seen from household perspectives. Figure 2 shows that per capita out-of-pocket expenditures on health rose from 2002 to 2020 by 3.9% in high-income economies, while a constant decrease is evident in low-income countries throughout the cited period. Thus, considering the theoretical assumptions that good governance ensures the integration of various macroeconomic strands, boosting economic performance and productivity through human capital well-being [7], it is of importance to delve more explicitly into its link with contemporary health expenditures. Indeed, the penetration of healthcare, availability, and accessibility of people to quality healthcare services are the key dimensions of human well-being and have direct effects on human capital development, reduction of mortality rates, and contemporary living standards. On the other hand, for instance, it is also well-evident in low- and middle-income countries that the healthcare sector falls short of providing the desired quality of healthcare services to people due to low institutional quality arising from low regulatory quality, extensive corruption, low efficacy of government, absence of the absolute rule of law, political instability in some instances [8], and weak accountability [9]. Thus, low governance efficacy not only traps people in losing their money; it also perpetuates the existing rampant corruption in the administration of healthcare services, limits accessibility to healthcare, increases unnecessary patients’ costs, deprives poor people of essential healthcare services, and thereby significantly impacts the overall social and economic development of a country [10]. Two empirical strands, as developed throughout recent decades, explain the function of health expenditures, such as the income view and the governance view of healthcare. The former has been critically analyzed by Yang et al. [11], Wu et al. [12], Bloom et al. [13], Bilgili et al. [14], Chireshe and Matthew [15], Raghupathi and Raghupathi [16], and many others, who confirm that health expenditure is positively connected with economic growth, implying that the higher the per capita income, the higher the health expenditures will be, whereas the governance view of healthcare has received little attention from scholars.

Therefore, the present study primarily aims to explicitly delve into the governance view of healthcare, using appropriate econometric techniques and large panel dataset to provide consistent results from a global perspective. In doing so, it is important to lead the discussion by formulating three key questions, among others. First, do health expenditures move together with good governance in the long run? Second, regardless of the income-level categories of the economies, does good governance have positive effects on health expenditures that correspond to the connotation of “governance for health”? Third, are the effects of good governance on health expenditures non-monotonic and vary both in terms of duration and income-level classifications of the economies? Providing consistent and accurate answers to these questions will not only fill the existing gaps in the literature but will also help policymakers understand the significant health policy implications of good governance from a different perspective.

The study’s novelty incorporates the determination of the impacts of good governance on health expenditures as a new step in the existing literature, and its contribution can be outlined as follows: First, it innovatively constructs a comprehensive composite governance index (CGI) under three key governance dimensions to capture the extensive effects of CGI on government health expenditures and per capita out-of-pocket health expenditures. In addition to highlighting the confounded results presented by most of the recent studies, it builds a new foundation in the empirical literature of governance for health through the construction of CGI that addresses the over-specification errors and encourages future studies to build upon it. Second, despite analyzing a panel of 180 countries, the study highlights serious policy shifts from income-view to governance-view by statistical confirmation of the non-monotonic and swift response of health expenditures to good governance both at income-level and global perspectives. It emphasizes that, despite the fact that health expenditures move with growth, good governance is critical to the long-term stabilization of this association.

The remaining parts of this article are organized as follows: Section two reviews the literature. Section three explains the data, variables, and key measurement issues. Section four explains the conceptual framework and the econometric techniques used to analyze the data. Section five presents the results. Section six provides a comparative discussion. Section seven concludes the study.

## Literature review

The literature documents several studies discussing the effects of good governance on health expenditures, providing mixed results. For instance, Farag [18] in low- and middle-income economies, Boz [19] in Turkey, Hilaire [20] in African countries, Kim and Lane [21], and Ahmad and Hasan [22] in Malaysia accentuate the effective role of good governance in the administration of health expenditures. Aljunid [23] explores the effects of governance on healthcare in Asia, using both empirical datasets and case studies about health expenditures, human capital, the distribution of health facilities, and utilization rates. They noticed that differences in healthcare utilization are primarily explained by quality of governance. Further, Radin [24] employed datasets from two surveys for 2007 and 2009 in Croatia to test the effects of healthcare corruption on public trust in the healthcare system. In 2007, the author discovered that corruption in healthcare services negatively impacts public trust in the healthcare system, whereas the results in 2009 were found to be insignificant. Lazarova and Ilaria [25] explored the effects of governance in 112 states and found that in countries below a certain threshold level, income is a significant determinant of healthcare, while in countries above a certain threshold level, governance significantly affects the outcome of healthcare. Likewise, Ouedraogo et al. [26] investigated the determinantal impact of institutional dimensions on healthcare outcomes in Sub-Saharan Africa. They found that governance is a key dimension that enhances the outcome of the healthcare system. Bovenkamp et al. [27] explored the effects of institutional quality layering on healthcare administration in Germany and employed market-based system analysis. They argued that using institutional quality arrangements affects healthcare quality and that hospitals need to deal with certain policy reforms to rectify the incremental change in the quality of healthcare demand. Dhrifi [28] examined the effects of public health expenditures on children’s health consequences, linked to the role of institutional quality in developed economies. The authors employed a two-step system generalized method of moment (GMM) and found that health expenditures are only significant in affecting children’s healthcare status in high-income economies, while it was found to be insignificant in low-, lower-middle-, and upper middle-income countries. They also indicated that institutional quality is a significant predictor in explaining the enhancement of healthcare system efficacy.

Furthermore, Rehmat et al. [29] investigated the impact of good governance on health expenditures in 105 countries. Their findings indicated that good governance has positive impacts on the population’s healthcare outcomes, increases life expectancy, and decreases the child mortality rate. They also found that economic growth, population density, and physicians’ practices have positive effects on life expectancy. Rajkumar and Vinaya [30] examined the links between governance, health expenditures, and health outcomes. They claim that the quality of governance largely explains the differences in health expenditures, lowering child mortality rates, enhancing school enrollment ratio, and increasing the efficacy of healthcare outcomes in countries exhibiting a higher quality of governance. Klomp and Jakob [31] analyzed the impact of the political system and its stability on healthcare using factor analysis and structural equation models. The authors employed national health indicators and economic and demographical variables. Their findings suggest that democracy has positive effects, while political instability has negative impacts on an individual’s healthcare status. Luca et al. [32] evaluated the effects of institutional quality on the provision of healthcare in Italy. They found that an increase of one standard deviation in institutional quality leads to the cesarean rate decreasing by ten basis points, showing that institutional quality enhances the healthcare outcome in Italy’s hospitals. Sharma et al. [33] examined the impact of the quality of economic institutions on health expenditures in European Union countries. They observed that an improvement in institutional quality has positive effects on the subject, showing that an efficient legal system and regulatory quality are the most effective indicators of the overall health outcomes.

In sum, an in-depth review of the existing literature demonstrates that, despite having many studies that explored the effects of good governance on health expenditures and significantly contributed to advance contemporary body of knowledge, most of the results presented by them might be confounded for two key reasons. First, while an exception is given to the work of Lazarova and Ilaria [25], who employed a quasi-governance index, almost all others are confronted with omitted variable bias. Second, the incorporation of perplexing governance proxies led to muddled policy implications. More importantly, prior literature reveals a scarcity, if not a complete absence, of studies that explicitly highlight the non-monotonic behavior of good governance on health expenditures, translated by income level rather than economic development classifications. With an exception to the work of Farag [18], who only covered low- and middle-income countries, global perspective has been totally ignored in the literature about the subject matter. Thus, to address these gaps, the study develops four new hypotheses, as follows: ***Hyp-1***: Good governance moves together with health expenditures (government and per capita out-of-pocket) in the long run; ***Hyp-2***: Good goverannce positively explains health expenditures both in the short and long run; ***Hyp-3***: The effects of good governance are non-monotonic and vary according to the countries’ income level; and ***Hyp-4***: Good governance has significant causal effects on health expenditures.

## Data and variables

### Data

This article employs annual balanced panel datasets for 144 countries spanning from 2002 to 2020. The selection of the data period was primarily conditional on its availability for the recipient countries included in our panel. For comparative analysis and extracting specific policy implications about the subject, we first employ a full sample (144 countries) and then use the World Bank’s economic classifications [34] to split the panels into high-income (49 countries), middle-income (40 countries), and low-income (55 countries) countries (see Appendix A of the Supplementary information (SI) for a complete list of countries).

### Selection of variabes

With reference to the primary objectives of the present study and to capture the precise impacts of good governance on health expenditures, the study constructs a comprehensive composite governance index (*CGI*) using six measures of Worldwide Governance Indicators. These measures include voice and accountability (*VoA*), political stability (*PoS*), government effectiveness (*GeF*), regulatory quality (*ReQ*), control of corruption (*CoC*), and the rule of law (*RoL*). The indicators are expressed in percentile ranks ranging from 0 (low) to 100 (high). For *CGI* construction, the study follows similar statistical methodology proposed by Sarma [35]. This method has several preferences over common index construction techniques and has recently gained empirical prominence in prior literature for generating both aggregate and dimensional indices [36], [37] in various macro- and socio-economic studies. Appendix B of the SI explains the construction methodology in detail. The choice of other variables is based on the conceptual background of the study and recent empirical literature. Consistent with studies by Wang et al. [38], Hameed et al. [39], and Rahman and Alam [40], government health expenditures (*GHE*, % of *GDP*) is used as the dependent variable. *GHE* includes all expenditures, both public and private, for the provision of health services, cost-bearing actions for family planning, nutrition, and emergency aid [41]. Although *GHE* measures contemporary healthcare status at the macro-level, the study employs per capita out-of-pocket expenditures on healthcare (*OPEH*) as another dependent variable to highlight the effects of *CGI* on the direct outlays by households. *OPEH* includes gratuities and in-kind payments to health practitioners and suppliers of medicines, therapeutic appliances, and other goods and services whose primary objectives are to contribute to the restoration or improvement of the individual’s health status [42]. Furthermore, considering the importance of endogenous variables, the study controls the effects of several predictors on the subject. They include the crude death rate (*CDR*), expressed as the number of deaths per 1,000 people; the child mortality rate (*CMR*), expressed as the number of deaths of children under 5 years old; and the age dependency ratio (*ADR*), expressed as the number of working people younger than 15 and older than 64 years old. Moreover, following prior studies (see, for instance, [43], [44]), the study employs tax revenue (*TAR*, % of *GDP*) to capture its effects on contemporary *GHE* and *OPEH*. While economic performance postulates direct effects on the subject [45], the study controls for the effects of *GDP* growth (annual%) on *GHE* and the effects of per capita *GDP* (*PCGDP*, constant 2015 US$) on *OPEH*.

### Source of compilation

We screened the available reliable sources to compile the required datasets for the present study. The datasets for *HE, CMR, GDPG, PCGDP, CDR*, and *ADR* were compiled from the World Development Indicators (WDI), sources that are relevant to the World Bank Group [6]. The datasets for governance indicators such as *VoA*, *CoC*, *RoL*, *ReQ*, *PoS*, and *GeF* come from the Worldwide Governance Indicators developed by Kaufmann and Kraay [46]. Finally, the data for *OPEH* has been compiled from the World Health Organization [17].

## Methodology

### Conceptual framework

In light of the governance-healthcare perspective, ideas about how countries address the determinants of healthcare are rapidly shifting, due to which two initial concepts have emerged. The first one emphasizes improving the output of healthcare systems through promoting the efficacy of contemporary governance, which is known as sector-specific governance [47]. The second concept promotes the synergistic triangle actions of private, public, and citizen sectors for a common social interest, known as the governance for health (say, comprehensive governance) [48]. Due to this imperative macroeconomic factor, comprehensive governance requires an increasing level of engagement from public organizations, societies, business firms, and citizens to achieve its fundamental objectives. As defined by the World Health Organization [49], health is essential to well-being and is a human right, which requires equity and social justice, gaining traction as an important component of society, resulting in economic prosperity, environmental sustainability, and social inclusion [50]. Comprehensive governance ensures that these interests are protected through the exercise of effective controls in utilizing the avaibale resources for the benefit of a country’s nation [51]. Pursuant to that, it gains ground on how to measure the efficacy of the so-called good governance. However, Kaufmann et al. [52] conceptualized the conduits to measure the efficacy of governance by six governance indicators, Acemoglu and Robinson [53] and Greif [54] have emphasized the social elements of these indicators to maintain the significance of their social comprehensiveness. Theories suggest that comprehensive governance plays a vital role in governance for health when formulating and enacting policies to encourage a participatory development viewpoint. Therefore, good governance increases people’s agency in the sense of the triangle engagement of private, public, and citizen sectors to actively engage in, plan for, and implement policies based on their development priorities and needs [55]. Having said so, we empirically conceptualize the study using the notion of governance for health and proceed with model specifications.

### Model specification

Pursuant to the connotation of “governance for health,” this study argues that both government health expenditures (*GHE*) and out-of-pocket expenditures on healthcare (*OPEH*) are significantly influenced by the practice of good governance at macro-levels, regardless of the size and economic structure of the underlying economies. Therefore, substantiating the exogeneity of health expenditures by good governance, the study specifies the following long-run linear multivariate panel models:

1 $$\begin{gathered} GH{E_{it}}={\delta _i}+{\eta _1}CG{I_{it}}+{\eta _2}GDP{G_{it}}+{\eta _3}CD{R_{it}}\,+{\eta _4}CM{R_{it}} \hfill \\ \,\,\,\,\,\,\,\,\,\,\,\,\,\,\,\,\,\,\,\,\,\,\,\,\,\,\,+{\eta _5}AD{R_{it}}+{\eta _6}TA{R_{it}}+{\eta _7}{\zeta _{it}}+{u_{it}} \hfill \\ \end{gathered}$$

where ${\delta _i}=$intercept, ${\eta _1}\,\,{\text{to}}\,\,{\eta _6}=$long-run parameters, ${\eta _7}=$coefficient of the dummy variable $({\zeta _{it}})$used to capture the effects of any structural breaks, $i=1,2,3,\ldots,=N,\,\,t=1,2,3,\ldots,=T,\,\,\,{\text{and}}\,\,{u_{it}}=$error term. Equation (1) assumes that, in the long run, *GHE* is influenced by *CGI* at a macro-level and that the effects of *CGI* vary due to the income-level of the underlying panel. Due to the argument, the signs of the coefficients are expected to be ${\eta _1},\,\,{\eta _2},\,\,{\eta _5},\,\,{\text{and}}\,\,{\eta _6}\,=(+)$and ${\eta _3},\,\,\,{\eta _4}=( - ).$ Further, the study attempts to examine the effects of *CGI* on *OPEH* across the recipient panels. Thus, having all other vectors and variables similar to Eq. (1), except for the *PCGDP*, the following long-run multivariate panel equation is specificed:

2 $$\begin{gathered} OPE{H_{it}}={\delta _i}+{\eta _1}CG{I_{it}}+{\eta _2}PCDP{G_{it}}+{\eta _3}CD{R_{it}}\,+{\eta _4}CM{R_{it}} \hfill \\ \,\,\,\,\,\,\,\,\,\,\,\,\,\,\,\,\,\,\,\,\,\,\,\,\,\,\,\,\,\,+{\eta _5}AD{R_{it}}+{\eta _6}TA{R_{it}}+{\eta _7}{\zeta _{it}}+{u_{it}} \hfill \\ \end{gathered}$$

**Fig. 3:**
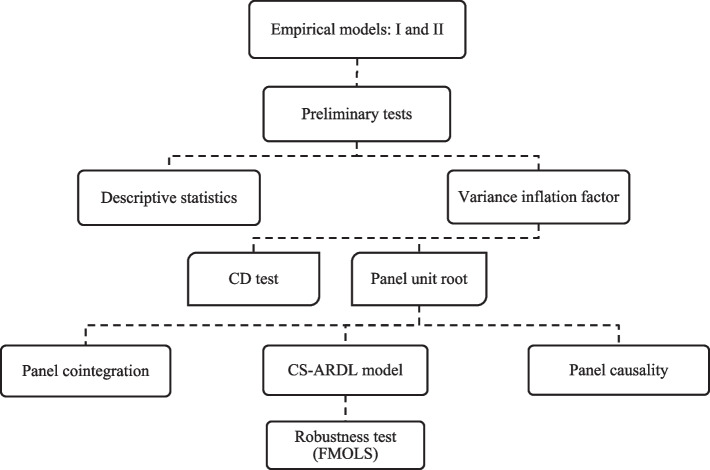
Estimation procedures.  Source: Authors’s creation

Moreover, for well-presented methods and results, the study follows the Strengthening the Reporting of Observational Studies in Epidemiology (STROBE) guidelines [56]. Thus, Fig. 3 explains the step-wise procedures that are carried out to estimate Eqs. 1 and 2 (say, models I and II).

### Estimation techniques

The existing literature offers numerous techniques for panel data analysis. It includes fixed effects (FE), random effects (RE), pooled ordinary least squares (POLS), autoregressive distributed lags (ARDL), and the generalized method of moment (GMM). These methods would be inconsistent and provide inefficient results in the presence of cross-sectional dependence (CD) among countries [57]. For instance, FE, RE, and the GMM models are used to correct for panel heterogenity issues in cases of small T and large N [58], but they are unable to capture the CD among panels. In real-life examples, panel data is generally subject to CD. It is obvious that due to common economic structures, common consumption behavior, international trade, and human capital mobility, CD exists among countries. Confirming the existence of CD by the rejected null of cross-sectional independence among the units through Pesaran’s [59] CD test, the study employs the cross-sectionally augmented autoregressive distributed lags (CS-ARDL) model of Chudik and Pesaran [60]. The CS-ARDL model augments the ARDL with a linear combination of the cross-sectional averages of the predictors to rectify the CD in the error term of the model [61]. Moreover, the CS-ARDL model captures the unobservable factors that are augmented to estimate the long-run effects in the regression. It also regards the 1-year lag period of the dependent variable as a weakly exogenous indicator within the error-correcting process [62]. For estimation, the CS-ARDL model utilizes both mean group (MG) and pooled mean group (PMG) estimators. However, we do not aim to outline the preference of the PMG over MG estimators (see [58] for technical review), but due to empirical facts—that is, if the long-run coefficients are similar across units, the PMG estimator is efficient and consistent [63]. Moreover, the MG estimator estimates biased and inconsistent coefficients in the presence of CD [64]. In this faith, the study specifies the CS-ARDL model as:

3 $$\begin{gathered} \Delta {Y_{it}}={\varphi _i}+{\alpha _i}\left( {{Y_{it - 1}} - \lambda _{i}^{\prime }{X_{it - 1}}+\alpha _{i}^{{ - 1}}{\eta _i}{{\bar {Y}}_t}+\alpha _{i}^{{ - 1}}\phi _{i}^{\prime }{{\bar {X}}_t}} \right) \hfill \\ \,\,\,\,\,\,\,\,\,\,\,\,\,\,\,\,\,\,\,\,\,\,+\sum\limits_{{j=1}}^{{v - 1}} {{\delta _{ij}}\Delta } {Y_{it - j}}+\sum\limits_{{j=0}}^{{u - 1}} {{\vartheta _{ij}}\Delta } {X_{it - j}}+\sum\limits_{{j=1}}^{{v - 1}} {{\varsigma _{ik}}\Delta } {{\bar {Y}}_{it - j}}+\sum\limits_{{j=0}}^{{u - 1}} {{\xi _{ik}}\Delta } {{\bar {X}}_{it - j}}+{\gamma _i}{\zeta _{it}}+{u_{it}} \hfill \\ \end{gathered}$$

where ${Y_{it}}=GH{E_{it}}\,{\text{and}}\,\,OPE{H_{it}}$ for unit *i* at time *t*, ${X_{it}}=5 \times 1$vector of the explanatory variables, $v=$lag operator of of the dependent variables, $u=$lag operator of the explanatory variables, $\lambda =$long-run coefficients, $\vartheta =$short-run coefficients, ${\bar {Y}_t}=$cross-sectional averages of the dependent variables, ${\bar {X}_t}=$cross-sectional averages of the explanatory variables, and ${\gamma _i}=$referes to the long-run coefficient of the dummy variable $({\zeta _{it}})$to capture the effects of structural breaks. Though conventional panel models ignore the presence of cross-sectional correlations, Eq. (3) rectifies the CD and estimates consistent coefficients [65]. Equation (3) also computes both short- and long-run coefficients. To test the robustness of the estimated long-run coefficients obtained from Eq. (3), the study employs the fully modified ordinary least squares (FMOLS) method of Pedroni [66], which corrects the serial correlation and panel endogeneity issues. The FMOLS model with asymptotic distribution can be expressed as:

4 $$\varphi _{{NT}}^{*} - \varphi =\left( {\sum\limits_{{i=1}}^{{v - 1}} {L_{{22i}}^{{ - 2}}} \sum\limits_{{i=1}}^{{u - 1}} {{{\left( {{y_{it}} - {{\bar {y}}_{it}}} \right)}^2}} } \right)\,\sum\limits_{{i=1}}^{{v - 1}} {L_{{11i}}^{{ - 1}}L_{{22i}}^{{ - 1}}} \left( {\sum\limits_{{i=1}}^{{v - 1}} {\left( {{y_{it}} - {{\bar {y}}_i}} \right)\vartheta _{{it}}^{*} - T{{\overset{\lower0.5em\hbox{$\smash{\scriptscriptstyle\frown}$}}{\lambda } }_i}} } \right)$$

where ${\vartheta }_{it}^{*}={\vartheta }_{it}-\left({\widehat{L}}_{21i} \div {\widehat{L}}_{22i}\right)\varDelta {y}_{it}$, ${\widehat{\lambda }}_{i}={\widehat{\xi }}_{21i}{\widehat{\psi }}_{21i}^{0}-\left({\widehat{L}}_{21i} \div {\widehat{L}}_{22i}\right)\left({\widehat{\lambda }}_{22i}+{\widehat{\psi }}_{22i}^{0}\right)$, and ${\widehat{L}}_{i}$ refers to the sample estimator of ${L}_{i}$ presenting the lower triangulation of ${\widehat{\psi }}_{i}$. To extend the analysis, it is important to explore the causal links between the *CGI*, *HE*, *OPEH*, and the control variables. In this faith, the study employs the panel causality test of Dumitrescu-Hurlin [67], which is rational in the presence of CD and is specified as:

5 $${Y_{it}}={\varphi _i}+\sum\nolimits_{{i=1}}^{k} {\eta _{i}^{{(k)}}{Y_{it - k}}} +\sum\nolimits_{{i=1}}^{k} {\xi _{i}^{{(k)}}{X_{it - k}}} +{\varepsilon _{it}}$$

6 $$Hyp=\left\{ \begin{gathered} {\xi _{i=0}}{\forall _i}=1,2,3,\ldots.,=N \hfill \\ {\xi _{i \ne 0}}{\forall _i}={N_1}+1,{N_2}+2,{N_3}+3,\ldots.,=N \hfill \\ \end{gathered} \right.$$

where ${\varphi _i}=$intercept, $\eta _{i}^{k}=$coefficient of *GHE* and *OPEH*, $\xi _{1}^{k},\ldots,\xi _{n}^{k}=$coefficients of the explanatory variables, $K\varepsilon {N^+}(K\varepsilon {N^ \times })=$constant, and ${\varepsilon _{it}}=$error-term. Equation (5) tests the null of no panel Granger causality between the variables vs. its alternative hypothesis. Both null and alternative hypotheses are presented in (6). Equation (5) assumes that the individual effects are constant when using different lags across units, but the coefficients of the slope and lag parameters vary across countries. All estimations are carried out using STATA-17/BE, R-Progamming, Eviews-11, and OriginLab-2023 software packages.

## Results

### Summary statistics

The analysis begins with some descriptive statistics (Table 1) to highlight important trends in the data. The results show that the mean value of *GHE* is 7.09% in the full sample, while it is 7.206% in the high-income, 6.211% in the middle-income, and 5.356% in the low-income panels, respectively. The mean value of *OPEH* stands at 33.716% of the GDP in the full sample, whereas it rounds to 31.961%, 33.241%, and 35.8% in the high-, middle-, and low-income panels, respectively. Further, the mean value of *CGI* is 0.731 in the full sample, 0.771 in the high-income panel, 0.456 in the middle-income panel, and 0.443 in the low-income panel, respectively. Irrespective of the control variables, the results indicate that *GHE, OPEH*, and *CGI* have an increasing trend, while *GHE* in the middle- and low-income panels exhibits weak volatility. Moreover, *CGI* also shows an improvement over time in both the middle- and low-income panels. Additionally, the study explores the multicollinearity among the variables of interest using the variance inflation factor (VIF) method. VIF has been computed as a post-estimation of the pooled OLS technique. The results are reported in Table 1 and demonstrate that VIF values are below the threshold level of 10 across all panels. Thus, it concludes that the variables do not exhibit any perfect or extreme multicollinearity.

### Optimal lag length

To accurately estimate the subsequent regressions, it is important to determine the optimal lag length. Empirically, adding more lags causes observations to be lost, while using a lower number of lags leads to an estimation that ignores detecting the dependence of variables on their past values. Thus, the study estimates the Akaike information criterion (AIC), Hannan-Quinn information criterion (HIC), and Schwarz information criterion (SIC) methods using the unrestricted vector autoregressive environment with the “*VARSOC*” command for the variables of interest employed in models (1) and (2). Altogether, the results suggest using one lag for panel data analysis.

**Table 1:** Descriptive statistics

Tests	*GHE*	*OPEH*	*GDPG*	*PCGDP*	*ADR*	*CMR*	*CDR*	*CGI*	*TAR*
***Full sample***									
Mean	6.219	233.921	5.070	8,628.31	60.524	38.394	8.332	0.731	16.739
Standard Deviation	2.500	11.991	6.011	3,299.13	18.848	41.015	3.207	0.567	7.668
Minimum	1.263	144.659	-3.338	255.100	16.172	1.800	0.795	0.213	0.079
Maximum	20.413	334.618	6.826	11,037.23	111.196	214.8	20.884	0.837	47.661
VIF			4.68	4.22	4.09	3.68	3.01	2.88	2.67
***High-income panel***									
Mean	7.206	657.566	1.991	35,874.98	47.486	8.543	7.860	0.771	17.192
Standard Deviation	2.701	31.428	4.575	27,921.92	9.023	8.342	2.899	0.711	6.864
Minimum	1.532	451.729	-4.782	4,125.33	16.172	1.945	0.816	0.715	0.586
Maximum	18.815	885.177	6.170	112,417.9	94.035	72.611	15.900	0.968	48.563
VIF			5.01	4.98	5.46	6.17	4.65	3.44	4.28
***Middle-income***									
Mean	6.211	158.045	3.339	6,343.945	54.747	24.500	7.704	0.546	16.353
Standard Deviation	2.120	9.409	7.117	2,601.32	10.745	19.881	3.084	0.449	5.918
Minimum	1.263	87.982	-5.338	1,527.93	35.399	2.640	2.718	0.408	0.420
Maximum	12.814	225.406	6.826	14,222.50	88.608	133.12	18.00	0.802	37.612
VIF			3.39	3.15	3.06	2.45	2.39	2.02	1.98
***Low-income panel***									
Mean	5.356	43.024	3.985	1,479.937	78.454	74.403	9.270	0.443	16.549
Standard Deviation	2.230	1.999	5.139	996.638	17.035	42.344	3.380	0.506	9.340
Minimum	1.701	23.947	-4.082	255.100	39.125	7.000	3.670	0.213	0.079
Maximum	20.413	51.430	10.629	4,495.710	111.196	214.80	20.884	0.565	27.108
VIF			4.14	3.87	3.62	2.99	2.26	2.19	1.68

Source: Authors’ estimations

### Cross-sectional dependence test

Due to world prices, common socioeconomic factors, similar technological advancements, and common trade and consumption behavior, panel data may exhibit cross-sectional dependence (CD), whether the predictors are correlated or not. To highlight this issue in the present analysis, Pesaran’s [59] CD test has been computed, and the results are reported in Table 2. The results indicate that the ARD and CDR are statistically insignificant to reject the null of cross-sectional independence across all the panels, while the remaining variables reject the null at a 1% significant level. Empirically, the existence of CD imparts serious bias problems in estimating the coefficients via common panel techniques; only adjustments are insufficient in the error term, as in [68].

**Table 2:** Cross-sectional dependence test results

Variables	Full sample		High-income		Middle-income		Low-income	
	CD-test	*p*-value	CD-test	*p*-value	CD-test	*p*-value	CD-test	*p*-value
*GHE*	61.26***	0.000	18.21***	0.000	35.45***	0.000	10.67***	0.000
*OPEH*	57.66***	0.000	33.47***	0.000	17.21***	0.000	49.02***	0.000
*CGI*	18.69***	0.000	10.33***	0.000	15.00***	0.000	21.12***	0.000
*GDPG*	163.63***	0.000	4.81***	0.000	4.33***	0.000	8.16***	0.000
*PCGDP*	217.80***	0.000	3.99***	0.000	110.27	0.000	88.32***	0.000
*ADR*	1.84	0.210	1.04	0.550	0.98***	0.525	1.12	0.475
*CMR*	48.47***	0.000	18.92***	0.000	42.19***	0.000	11.37***	0.000
*CDR*	1.40	0.325	0.91	0.612	1.16	0.375	0.77	0.612
*TAR*	3.15***	0.002	55.47***	0.000	18.99***	0.000	20.36***	0.000

Source: Authors’ estimations

Notes: *** indicates significance at a 1% level

### Panel unit root test

In the presence of CD, the study uses the second-generation panel unit root test of Pesaran’s [69] and Ditzen et al.’s [70] structural break techniques. The results are presented in Table 3. It shows that for the rejected null of non-stationarity in the full sample, *GHE, OPEH, CGI, GDPG*, and *CMR* are significant at a 1% level, while the remaining variables can only reject the null after taking their first difference. In the panel of high-income economies, almost similar results are achieved except for *PCGDP*, which is a level-stationary variable. In the middle-income panel, *OPEH*, *CGI*, *ADR*, and *CMR* are significant for rejecting the null at the level, whereas other predictors are found to be first difference stationary. Finally, in the low-income panel, *GHE, OPEH, CGI, GDPG, PCGDP*, and *TAR* are found to be significant for rejecting the null of non-stationarity, whereas the remaining variables exhibit significance after the first difference. Besides, the results also confirm the existence of structural breaks in different years led by data trends. The results provide important insights, leading the study to explore any cointegration between the variables.

**Table 3:** CIPS panel unit root results

Variables	Full sample			High-income panel		
	Level	First-difference	Break	Level	First-difference	Break
*GHE*	-3.08***	-3.92***	2004	-4.11***	-4.67***	2004
*OPEH*	-2.28***	-3.80***	2006	-4.39***	-4.98***	2009
*CGI*	-3.25***	-5.09***	2011	-3.48***	-4.10***	2003
*GDPG*	-2.92***	-4.71***	2008	-4.00***	-4.29***	2008
*PCGDP*	-1.34	-2.66***	2008	-2.55***	-3.12***	2008
*ADR*	-1.52	-2.41***	2012	-0.99	-2.26***	2011
*CMR*	-2.42***	-2.51***	2012	-2.81***	-3.25***	2006
*CDR*	-1.19	-2.66***	2005	-1.32	-2.49***	2003
*TAR*	-1.25	-2.87***	2009	-1.16	-2.67***	2007
	Middle-income panel			Low-income panel		
*GHE*	-1.82	-2.74***	2007	-3.21***	-3.98***	2005
*OPEH*	-2.33***	-2.89***	2007	-4.45***	-4.99***	2008
*CGI*	-4.13***	-4.87***	2015	-2.31***	-3.05***	2003
*GDPG*	-1.10	-2.29***	2010	-3.16***	-3.66***	2009
*PCGDP*	-0.99	-2.21***	2009	-4.22***	-4.78***	2009
*ADR*	-1.11***	-2.37***	2011	-1.14	-2.27***	2012
*CMR*	-4.18***	-5.00***	2012	-1.49	-2.67***	2004
*CDR*	-0.67	-2.19***	2007	-1.01	-2.21***	2005
*TAR*	-1.44	-2.63***	2016	-0.66***	-2.19***	2008
CIPS critical values	1%	5%	10%			
	-2.14	-2.04	-1.99			

Source: Authors’ estimations

Notes: *** indicates significance at a 1% level

### Cointegration analysis

Based on our primary objectives, it is crucial to delve into the long-run association between the variables. Thus, the study employs Westerlund’s [71] cointegration model for heterogeneous panels. This method is appropriate and produces consistent results in the presence of structural breaks and CD in the panels. Furthermore, it also employs the proposed panel cointegration test of Pedroni [72] for cross-validation. The results are presented in Table 4. Altogether, the results of Pedroni’s test are significant to reject the null of no cointegration across all panels, though they might be inconsistent in the presence of CD. Nevertheless, based on the outcome of Westerlund’s test, the results confirm the rejection of the null of no cointegration for all the panels across both models I and II.

**Table 4:** Panel cointegration results

Models estimated	*Westerlund’s test*	*Pedroni’s test*		
	Variance ratio	M.PP-stat.	PP-stat.	ADF-stat.
***Model I- Dependent variable: HE***				
Full sample	-10.33***	-3.49***	-3.26***	-3.61***
High-income panel	-5.67***	-4.33***	-3.44***	-3.82***
Middle-income panel	-4.12***	-4.21***	-3.90***	-3.98***
Low-income panel	-4.87***	-4.99**	-4.37***	-4.06***
***Model II-Dependent variable: OPEH***				
Full sample	-9.69***	-4.18***	-3.82***	-3.90***
High-income panel	-14.58***	-9.37***	-6.41***	-8.34***
Middle-income panel	-3.72***	-3.55***	-3.19***	-4.00***
Low-income panel	-4.17***	-3.61***	-3.15***	-3.92***

Source: Authors’ estimations

Notes: *** indicates significance at a 1% level. M.: Modified, PP: Phillips-Perron, ADF: Augmented Dickey-Fuller

### CS-ARDL estimation

Confirming the long-run association between the variables, the study proceeds to explore the effects of *CGI* on *GHE* (model I) and *OPEH* (model II) using the CS-ARDL (*u = 1, v = 1*) model. The results are reported in Tables 5 and 6. The estimation of the CS-ARDL model is in line with the primary objective of the study, that is, to provide a comparative analysis of the world’s performance viz-à-viz countries classified by income levels. For clarity, the study presents each panel’s results separately as follows:

**Table 5:** CS-ARDL estimates-model (I)

Model I-Dependent variable: GHE	Full sample		High-income		Middle-income		Low-income	
	Statistics	*p-value*	Statistics	*p-value*	Statistics	*p-value*	Statistics	*p-value*
***Short-run effects***								
CGI	1.37	0.420	0.81	0.325	0.61	0.410	0.55	0.625
GDPG	0.16***	0.000	0.12***	0.000	0.22***	0.002	0.19***	0.000
ADR	0.29*	0.067	0.14***	0.000	0.11***	0.000	0.16***	0.000
CMR	-0.34***	0.000	-0.17***	0.000	-0.18***	0.001	-0.15***	0.000
CDR	-0.26***	0.000	-0.35***	0.000	-0.28***	0.000	-0.21***	0.000
TAR	0.0071	0.725	0.003	0.965	0.0009	0.510	0.0017	0.119
Constant	-6.42***	0.000	-2.13***	0.000	-4.67***	0.000	-6.09***	0.000
***Long-run effects***								
CGI	0.85***	0.000	0.97***	0.000	0.68***	0.000	0.57***	0.000
GDPG	0.31***	0.000	0.18***	0.000	0.25***	0.000	0.18***	0.000
ADR	0.36***	0.000	0.20***	0.000	0.14***	0.000	0.33***	0.000
CMR	-0.42*	0.062	-0.24***	0.000	-0.19***	0.000	-0.22***	0.000
CDR	-0.28**	0.033	-0.41*	0.072	-0.35*	0.051	-0.27**	0.018
TAR	0.049**	0.047	0.026*	0.089	0.041**	0.027	0.046*	0.057
*ζ*_*it*_	0.0013	0.555	-0.008	0.410	-0.0009	0.625	-0.00003	0.720
***Robustness checks***								
R-squared	0.54		0.41		0.38		0.55	
F-statistics	44.98***	0.000	19.45***	0.000	10.33***	0.000	28.67***	0.000
CD-statistics	-1.016	0.325	-0.991	0.410	-1.67	0.310	-0.847	0.425
JB-normality	1.762	0.210	1.88	0.175	2.018	0.120	1.156	0.255

Source: Authors’ estimations

Notes: ***, **, and * indicate significance at 1%, 5%, and 10% levels, respectively

**Table 6:** CS-ARDL estimates-model (II)

Model I-Dependent variable: OPEH	Full sample		High-income		Middle-income		Low-income	
	Statistics	*p-value*	Statistics	*p-value*	Statistics	*p-value*	Statistics	*p-value*
***Short-run effects***								
CGI	0.046	0.000	0.012	0.620	0.071	0.425	0.72	0.240
PCDPG	0.69**	0.000	0.109***	0.000	0.84***	0.000	0.72***	0.000
ADR	0.027***	0.000	0.18***	0.000	0.109***	0.000	0.15***	0.000
CMR	-0.33***	0.000	-0.12*	0.077	0.20**	0.043	-0.17*	0.091
CDR	-0.28*	0.099	-0.32*	0.069	-0.30*	0.055	-0.22*	0.058
TAR	0.008	0.333	0.0004	0.815	0.0002	0.525	0.00015	0.444
Constant	-4.33***	0.000	-9.46***	0.000	-9.21***	0.000	-5.67***	0.008
***Long-run effects***								
CGI	0.61*	0.052	0.91***	0.001	0.28***	0.000	0.13***	0.000
PCDPG	0.82***	0.000	0.097***	0.000	0.99***	0.000	0.78***	0.000
ADR	0.033*	0.077	0.041***	0.000	0.51***	0.000	0.33***	0.000
CMR	-0.27***	0.000	-0.16***	0.000	-0.13***	0.000	-0.17**	0.036
CDR	-0.42***	0.009	-0.37***	0.000	-0.46***	0.000	-0.28***	0.000
TAR	0.0012*	0.081	0.007***	0.000	0.0013*	0.098	0.0009**	0.025
*ζ*_*it*_	-0.0671	0.965	-0.0044	0.880	-0.0009	0.245	-0.00016	0.710
***Robustness checks***								
R-squared	0.67		0.81		0.59		0.48	
F-statistics	8.33***	0.000	12.65***	0.000	7.94***	0.000	18.19***	0.000
CD-statistics	-0.67	0.450	-1.111	0.375	-1.099	0.320	-0.95	0.410
JB-normality	2.08	0.110	1.88	0.115	1.905	0.122	2.001	0.110

Source: Authors’ estimations

Notes: ***, **, and * indicate significance at 1%, 5%, and 10% levels, respectively

#### Full sample

For *CGI*, the results indicate that it is only significant to positively influence *GHE* and *OPEH* in the long run; short-run effects are found to be insignificant. A 1% increase in *CGI* causes *GHE* and *OPEH* to rise by 0.85% and 0.011%, respectively. The specificity of *CGI*’s long-term effects on *GHE* and *OPEH* is due to policy adjustments, implementation, the institutionalization of good governance practices, and the assessment of relevant policy outcomes. Moreover, the results show that *GDPG* (*PCGDP*) improves *GHE* (*OPEH*) by 0.16% (0.69%) and 0.31% (0.82%) in the short and long runs, respectively. It indicates that the growth at the macro-level has a comparatively lower power to explain *GHE* than that of per capita income, which explains *OPEH*. It is linked to facts. Lack of access to health insurance and free public health centers in rural areas in most of the developing economies causes individuals to bear health expenses.

The results also demonstrate that a 1% increase in *CMR* and *CDR* causes *GHE* and *OPEH* to decline by 0.34% (0.33%) and 0.26% (0.28%) in the short run and by 0.42% (0.27%) and 0.28% (0.42%) in the long run, respectively. The *ADR* is also found to have positive effects on both *GHE* and *OPEH*, while the *TAR* is only found to have a weak positive impact on the subject in the long run.

#### High-income panel

In the high-income panel, the results obtained from both models I and II indicate that *CGI* is substantial to influence *GHE* and *OPEH* in the long run, while the short-run effects are statistically insignificant. It indicates that a 1% increase in *CGI* causes *GHE* and *OPEH* to improve by 0.97% and 0.91%, respectively. In contrast to the full sample, the results indicate that *CGI* has a higher positive effect on both *GHE* and *OPEH*. It implies that governance establishments and higher-quality institutions are highly effective in improving contemporary *GHE* and *OPEH* in the high-income panel compared to those in the full sample. For the control variables, similar results were found. The findings show that *GDPG* has a significant positive impact on *GHE*. *PCGDP* is found to have a comparatively lower effect on *OPEH* in the high-income panel countries. This might be, again, due to the higher access of the population to health centers, health insurance, and well-developed establishments in high-income countries. Furthermore, *CMR* and *CDR* exert positive effects on both *GHE* and *OPEH*, while *ADR* negatively explains the subject. The *TAR* has only long-term positive effects on *GHE* and *OPEH*, with no short-term effects.

#### Middle-income panel

In the middle-income panel, the results indicate that *CGI* has only long-run effects on both *GHE* and *OPEH*. It shows that a 1% increase in *CGI* causes *GHE* (*OPEH*) to rise by 0.68% (0.28%) in the long run. Comparatively, the size of the effects of *CGI* in the high-income panel is higher than that of the middle-income panel, indicating that larger economies attract higher *CGI* effects. Moreover, the results show that *GDPG* (*PCGDP*) spurs *GHE* (*OPEH*) by 0.22% (0.84%) in the short run and 0.25% (0.99%) in the long run. *CMR* and *CDR* are found to negatively associated with *GHE* and *OPEH* in the short- and long-run, while the *ADR* is positively linked with both *GHE* and *OPEH*. Likewise, the *TAR* only has long-run weak positive impact on the subject, showing that it increases *HE* (*OPEH*) by 0.04% (0.0013%).

#### Low-income panel

Finally, the results for the low-income economies show that *CGI* is only significant to improve both *GHE* and *OPEH* in the long run by 0.57% and 0.13%, respectively. Again, it shows that, compared to the full, high- and middle-income countries, the health outcomes in low-income countries are less affected by *CGI*. However, the results show that while *GDPG* (*PCGDP*) is positively associated with *GHE* (OPEH), CMR and CDR have a negative influence on both *GHE* and *OPEH*. Specifically, a 1% increase in *GDPG* (*PCGDP*) improves *GHE* (*OPEH*) by 0.19% (0.15%) in the short run and 0.18% (0.78%) in the long run. For other control variables, similar results are found. For instance, while *ADR* has positive effects, *CDR* and *CMR* have negative impacts on both *GHE* and *OPEH* in the short and long run. Similarly, the *TAR* is found to have only long-run effects on both *HE* and *OPEH*. It shows that a 1% increase in *TAR* improves *GHE* (*OPEH*) by 0.046% (0.0009%) in the long run.

#### Robustness tests

For statistical validation of the results obtained from the CS-ARDL estimations reported in Tables 5 and 6, we adopted two approaches. First, we computed some important diagnostic checks and reported the results in the rear part of Tables 5 and 6. They indicate that the CS-ARDL model estimates have corrected the CD across all panels, and the residuals are normally distributed. Additionally, the estimates have taken the effects of structural breaks into account. The results indicate that the coefficients of the structural breaks (${\zeta }_{it})$ are insignificant to influence the GHE and OPEH in all recipient panels. Second, we estimated the FMOLS model to check the robustness of the long-run coefficients. The results reported in Table 7 indicate that the coefficients are robust and correspond to those of the estimations shown in Tables 5 and 6.

**Table 7:** FMOLS estimates

Variables	Full sample		High-income panel		Middle-income panel		Low-income panel	
	Statistics	*p-value*	Statistics	*p-value*	Statistics	*p-value*	Statistics	*p-value*
***Model I-dependent variable: GHE***								
CGI	0.66***	0.000	0.82***	0.000	0.91***	0.000	0.49***	0.000
GDPG	0.47***	0.000	0.31***	0.000	0.22***	0.000	0.45***	0.000
ADR	0.22***	0.000	0.28***	0.000	0.18***	0.000	0.25***	0.000
CMR	-0.88***	0.000	-0.29***	0.000	-0.11***	0.000	-0.13***	0.000
CDR	-0.19***	0.008	-0.36***	0.000	-0.30***	0.001	-0.29***	0.000
TAR	0.0049***	0.000	0.00016***	0.003	0.00026***	0.000	0.0004***	0.000
Constant	28.916***	0.000	33.412***	0.000	18.009***	0.000	10.682***	0.007
*ζ*_*it*_	-0.00089	0.277	-0.000021	0.418	-0.000096	0.850	-0.00067	0.650
***Post-estimations***								
R-squared	0.45		0.38		0.33		0.49	
Normality test	1.88	0.325	2.11*	0.099	0.94	0.520	1.98	0.111
***Model II-dependent variable: OPEH***								
CGI	0.44***	0.000	0.86***	0.000	0.23***	0.000	0.29***	0.000
PCGDP	0.67***	0.000	0.101***	0.000	0.84***	0.000	0.51***	0.000
ADR	0.11***	0.000	0.003***	0.000	0.67***	0.000	0.42***	0.000
CMR	-0.22***	0.000	-0.19***	0.000	-0.10***	0.000	-0.19***	0.000
CDR	-0.37***	0.000	-0.24***	0.000	-0.55***	0.000	-0.12***	0.000
TAR	0.003***	0.000	0.0006***	0.000	0.0003***	0.000	0.00011***	0.009
Constant	19.33***	0.000	21.45***	0.000	-6.77***	0.000	9.38***	0.000
*ζ*_*it*_	-0.0185	0.510	-0.0044	0.920	-0.0066	0.567	-0.0093	0.465
***Post-estimations***								
R-squared	0.47		0.52		0.38		0.50	
Normality test	0.87	0.510	1.87	0.325	0.92	0.475	1.32	0.425

Source: Authors’ estimations

Notes: *** indicates significance at a 1% level

### Panel causality test

To conclude the analysis, we compute the Dumitrescu-Hurlin [67] panel causality test and report the results in Table 8. The results show that, in all panels, CGI is significant at a 1% level to cause both *GHE* and *OPEH*. It also shows that in models I and II, *GDPG, PCGDP, CDR*, and *ADR* have a statistically significant causal nexus with *G*HE and *OPEH* in all recipient panels. Furthermore, the results indicate that CMR and TAR are insignificant in causing *GHE* and *OPEH* across all panels. The findings also do not lend statistical support for reverse causality from GHE and *OPEH* to the explanatory variables. Thus, they are not reported.

**Table 8:** Dumitrescu-Hurlin causality results

Causality direction	Full sample		High-income		Middle-income		Low-income	
	Z-stat.	*p-values*	Z-stat.	*p-value*	Z-stat.	*p-value*	Z-stat.	*p-value*
***Model I-dependent variable: GHE***								
CGI→GHE	4.13***	0.000	8.11***	0.000	4.16***	0.000	5.67***	0.000
GDPG→GHE	6.47***	0.000	3.43***	0.000	5.68***	0.000	7.22***	0.000
CMR→GHE	1.11	0.440	1.32	0.425	0.88	0.310	1.28	0.325
CDR→GHE	4.67***	0.000	4.33***	0.000	5.45***	0.000	2.19*	0.055
ADR→GHE	9.88***	0.000	6.36***	0.000	3.99***	0.000	5.41***	0.000
TAR→GHE	0.94	0.410	0.67	0.420	1.36	0.455	0.88	0.420
***Model II-dependent variable: OPEH***								
CGI→OPEH	3.99***	0.000	5.17***	0.000	8.04***	0.000	9.85***	0.000
PCGDP→OPEH	4.17***	0.000	2.82**	0.045	3.96***	0.000	9.14***	0.000
CMR→OPEH	0.58	0.555	1.24	0.440	0.46	0.630	1.39	0.450
CDR→OPEH	6.45***	0.000	8.19***	0.000	10.11***	0.000	7.82***	0.000
ADR→OPEH	2.98***	0.009	4.33***	0.000	6.87***	0.000	7.11***	0.000
TAR→OPEH	1.21	0.425	0.55	0.625	1.53	0.310	1.44	0.305

Source: Authors’ estimation

Notes: ***, **, and * indicate significance at 1%, 5%, and 10% levels, respectively

**Fig. 4:**
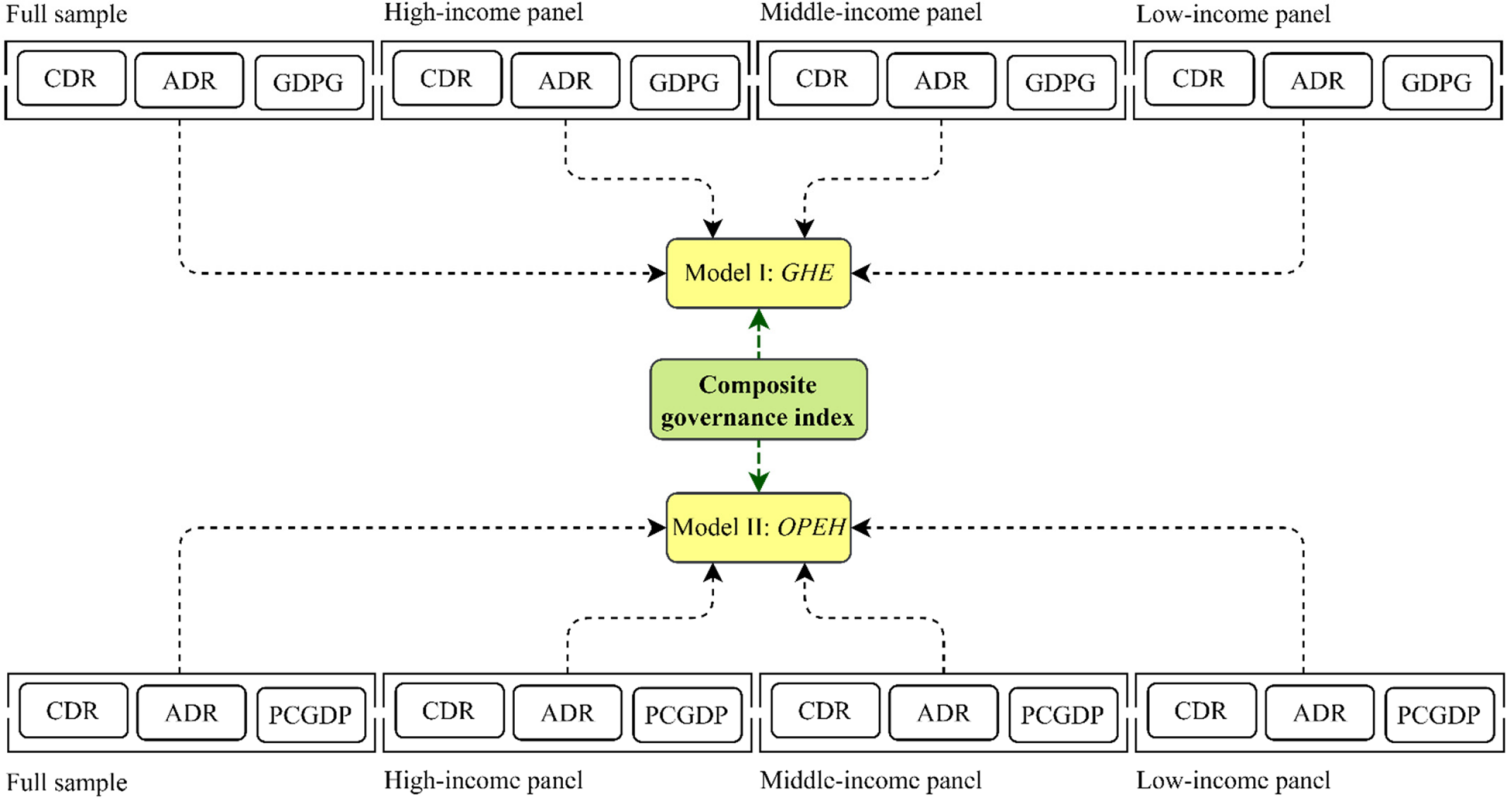
Overall panel causality results.  Source: Authors’ estimation

For brevity, we display the overall result of the panel causality for all panels in Fig. 4. The dashed lines indicate the significant causal relationships with corresponding *p-values* at 1%, 5%, and 10% running from *CGI* and explanatory to both *GHE* and *OPEH* (say, models I and II).

## Discussions

This study raised two key arguments. First, macro-level institutional quality (governance for health) viz-à-viz health sector-specific governance is more critical to explaining health outcomes. Second, the volatility of the effects of good government on health outcomes is translated by the income level of the underlying economies. The initial descriptive statistics (Table 1) highlight varying trends of *GHE* (government health expenditures), *OPEH* (per capita out-of-pocket expenditures on healthcare), and *CGI* (composite governance index) that sharply increase in the full sample and high-income panel, but they indicate comparatively weak volatility in the middle-income and low-income panels. The slow-shifting trend of *CGI* in middle- and low-income countries might be due to their level of commitment to practicing good governance or the lack of sufficient specificity of governance in their enacted policies [73]. The analysis determines that *CGI* has a long-run relationship with *GHE* and *OPEH* in all recipient panels (Table 4), showing evidence that *CGI* differently affects *GHE* and *OPEH* in the long run. The results are consistent with those of Atay et al. [74], Dhrifi [28], Rahman and Alam [75], and Zhang et al. [76], who also found cointegration between institutional quality and health outcome indicators.

Furthermore, the results obtained from the CS-ARDL model (Tables 5 and 6) clearly indicate the partial non-rejection of the second hypothesis. The results fail to provide short-run effects of *CGI* on both *GHE* and *OPEH*. This is truly literal: the slow-shifting trend of *CGI* takes a long time to exhibit effects on the subject. From an economic viewpoint, the effects of *CGI* on *GHE* and *OPEH* can be tracked through three key conduits: First, *CGI* is central to facilitating a stable environment to attract sound healthcare projects and efficiently channelize *GHE* and *OPEH* to extend coverage and outreach to more individuals. Second, it optimizes both *GHE* and *OPEH* through effective administrative interventions. Third, the *CGI*, which is built on three key dimensions such as accountability, transparency, and participation shows that effective engagement of the private, public, and citizen sectors leads to greater efficiency in increasing both *GHE* and *OPEH*. Thus, an increase in *GHE* and *OPEH* that is effectively governed results in the enhancement of a healthcare system’s outcome. The findings are consistent with those of Filmer and Pritchett [77], Farag et al. [18], Ibukun [78], Ahmad and Hasan [22], and Chireshe and Ocran [79], who found that institutional quality improves health outcomes and enhances the effectiveness of healthcare outcomes. Moreover, and interestingly, the results show that *CGI*’s coefficients are higher in high-income economies, experiencing a slow decline in middle-income and low-income countries. This implies that health outcome indicators are less sensitive to *CGI* in high-income countries but more sensitive in middle- and low-income economies. Therefore, it significantly favors the non-rejection of the third hypothesis.

Altogether, the results of the CS-ARDL model in all the panels confirm the positive effects of *PGDP* on *GHE* and *PCGDP* on *OPEH* in both the short- and long-term. Recent studies by Chaabouni et al. [80], Bozkurt [81], Zaidi and Saidi [82], Rahman and Alam [83], and Wang [84], also found that economic growth has positive impacts on health outcomes. Furthermore, the *ADR* is positively associated with both *GHE* and *OPEH* in all panels. This implies that higher *ADR* has a negative impact on many people’s earnings and savings—that is, increasing the cost of health insurance provided by employers not only reduces wages but may also force employers to replace skilled labor with semi-skilled or unskilled labor to bear lower wage payments to cover higher health insurance costs [85]. For the *CMR*, the findings reveal that it negatively effects both *GHE* and *OPEH* in all panels in the short- and long-runs. In an empirical sense, this negativity might be due to two key reasons. First, the limited availability of healthcare services both to urban and rural populations caused by either a limited number of healthcare centers or higher cost of healthcare services; and second, the limited accessibility of people to healthcare services provided both by private and public healthcare centers. The elasticities of the effects of the *CMR* in different income-level groups support this empirical notion. For instance, the effects are lower in high-income economies than in low-income countries, indicating that both the availability and accessibility dimensions of healthcare services are higher in high-income and lower in low-income economies. Maruthappu et al. [86] and Rahman et al. [87] discovered that reductions in health expenditures significantly increased *CMR*, whereas Ortega et al. [88] provided statistical evidence on the direct effects of good governance, proxied by government effectiveness on reducing *CMR* and healthcare inputs. Among all others, Kiross et al. [89] and Novignon and Lawanson [90] reflected almost similar findings for Sub-Saharan African countries. The *CDR* is also found to be negatively affecting both *GHE* and *OPEH* in all panels both in the short- and long-runs. Again, the negativity of *CDR* occurs when there is a significant imbalance between the birth rate and the *CDR*—that is, the death rate is higher than the birth rate. The results also show that the size of the effects is higher in high-income countries than in middle and low-income economies, indicating a real-life example of the suppression of the birth rate in high-income economies and a higher birth rate in low-income countries. Similar findings were presented in recent studies by Rahman et al. [91], Berger et al. [92], and Elola et al. [93]. Finally, the study tested the effects of *TAR* on both *GHE* and *OPEH*. The results demonstrate that *TAR*, though weak in effect size, is significant in improving health outcomes in the long run across all the recipient panels. Likewise, Behera and Dash [43] found that tax revenue is positively associated with health expenditures in Indian states’ context. In a bid to offer more insights, the study explored the causal links between *GHE*, *OPEH*, *CGI*, and other control variables (Fig. 3). The results indicate a strong causal relationship running from *CGI*, *GDPG*, *PCGDP*, *ADR*, and *CDR* to both *GHE* and *OPEH*, while *CMR* and *TAR* were found to be insignificant. It displays that there is a unidirectional causality running from the cited predictors to *G*HE and OPEH across all panels, whereas feedback response was not observed. These findings favor the acceptance of the fourth hypothesis. These results are partially supporting the findings of Ashiabi et al. [94], Owusu et al. [95], and Rana et al. [96], while the results are in contrast with those of Akinlo and Sulola [97].

In sum, the overall findings show that *CGI* is a significantly effective tool for a conservative enhancement of health expenditures both at macro- and individual-levels, resulting in improved healthcare coverage rather than bearing unnecessary costs that force *GHE* and *OPEH* to rise. The results also support the emerging notion of “governance for health” rather than health sector-specific governance due to its comprehension and the engagement of three key societal forces.

## Conclusions

This article examined the effects of good governance on health expenditures. It hypothesized that good governance is central to channeling the enhancement of healthcare services through effective interventional conduits. The study employed a large panel dataset to provide a comprehensive empirical image to support decision-making at macro-policy levels. The datasets contain 144 countries (full sample), classified into high-income (49 countries), middle-income (40 countries), and low-income (55 countries), and were collected from various reliable sources over the period from 2002 to 2020. To capture the extensive impact of good governance on health expenditures, the study constructed a composite governance index (*CGI*) under three key dimensions such as accountability, transparency, and participation. Considering the stationarity properties of the variables, the study used the CS-ARDL model to analyze the data.

The findings reveal that *CGI*—the key variable of interest—has significantly positive effects on *G*HE and OPEH in the long run, implying that *CGI* is an essential tool to improve health expenditures. An in-depth analysis also indicates three more important findings. First, it confirms that the effect of *CGI* on *G*HE and OPEH is partially dependent on the economic size of the countries. It substantively implies that the effects of *CGI* are lower (higher) in high-income (middle- and low-income) countries. Second, it implies that *G*HE and OPEH are highly sensitive to *CGI*, improve with the practice of good governance, and achieve stabilization through the steady and long-term implementation of good governance. Third, the findings also reveal that *GDPG*, *PCGDP*, *TAR*, and *ADR* increase both *GHE* and *OPEH*. It notes that *GDPG*, *PCGDP*, and *TAR* enhance the scope of healthcare outcomes, while *ADR* increases the cost burden of *GHE* and *OPEH* as a force majeure. Moreover, *CMR* and *CDR* are found to have significant negative impacts on the subject across all panels, both in the short and long runs. The study concluded with the test of causality and found that *CGI*, *PCGDP*, *GDPG*, *CDR*, and *ADR* have a unidirectional causal effect on *GHE* and *OPEH* with no feedback response, while *TAR* and *CMR* were insignificant.

### Policy implications

The findings entail several important policy implications that can be outlined as follows: First, good governance is found to be essential in effectively and conservatively increasing health expenditures both at macro- and micro-levels, resulting in the enhancement of the efficacy of healthcare services and the improvement of the population’s health status across the globe. Thus, regardless, it is imperative that governments attempt to formulate (revise) relevant policies for the health sector to support, promote, and institutionalize the practice of good governance dimensions. Second, given the importance of the multifaceted impact of good governance along with other macroeconomic indicators, it is important that governments attempt to enhance growth-inclusiveness to increase the health-input value of per capita expenditure to ensure greater health output, particularly in middle- and low-income countries. Third, healthcare is fundamental to human capital development. Considering the non-monotonic impact of good governance on health expenditures, it is necessary for low- and middle-income countries to recognize the important role of good governance in their sustainable development strategies via a strong commitment to channelize good governance as an important healthcare administration.

### Limitations

The present study suffers from one key limitation. Although the construction of *CGI* has empirically improved the incorporation of many explanatory variables into the health-governance model, it still suffers from the lack of a single health proxy as the outcome variable. Future studies may attempt to overcome this empirical shortcoming by constructing a health-inclusive index to assure the amalgamation of relevant healthcare dimensions into the so-called “composite health index.”.

### Supplementary Information

**Additional file 1.**

## Data Availability

The datasets for *OPEH, CMR, GDPG, PCGDP, CDR*, and *ADR* were compiled from WDI. The datasets for governance indicators such as *VoA*, *CoC*, *RoL*, *ReQ*, *PoS*, and *GeF* come from WGI and the data for *OPEH* has been compiled from WHO. All sources are publicly and freely available. The complete CGI constructed dataset will be made available upon request.

## Abbreviations

ADF: Augment Dickey and Fuller
ADR: Age dependency ratio
CD: Cross-sectional
CMR: Child mortality rate
CDR: Crude death rate
CGI: Composite governance index
CS-ARDL: Cross-sectionally augmented autoregressive distributed lags
GDPG: Gross domestic product growth
GHE: Government health expenditures
OPEH: Per capita out-of-pocket expenditures on healthcare
PCGDP: Per capita gross domestic product
PP: Phillips-Perron
WDI: World Development Indicators
WGI: Worldwide Governance Indicators
WHO: World Health Organization
