# Supplementary material for: A global perspective on the governance-health nexus
Source: BMC Health Serv Res. 2023 Nov 10;23:1235. doi: 10.1186/s12913-023-10261-9 (PMC10638824; doi:10.1186/s12913-023-10261-9)
Supplement: Supplementary file 1 — Additional file 1. [file 12913_2023_10261_MOESM1_ESM.docx]

**Supplementary material**

**Appendix A: List of countries**

Afghanistan, Angola, Argentina, Armenia, Australia, Austria, Azerbaijan, Bahamas, Bangladesh, Belarus, Belgium, Belize, Brunei Darussalam, Bulgaria, Burkina Faso, Burundi, Cabo Verde, Cameroon, Canada, Central African Republic, Chad, Chile, Colombia, Comoros, Congo, Dem. Rep., Congo, Rep., Costa Rica, Cote d'Ivoire, Croatia, Cuba, Cyprus, Czechia, Denmark, Dominica, Dominican Republic, Ecuador, Egypt, Arab Rep., El Salvador, Equatorial Guinea, Estonia, Eswatini, Ethiopia, Fiji, Finland, France, Gabon, Gambia, Georgia, Germany, Ghana, Greece, Greenland, Grenada, Guatemala, Guinea, Guinea-Bissau, Guyana, Haiti, Honduras, Hungary, Iceland, India, Iran, Islamic Rep., Iraq, Ireland, Israel, Italy, Jamaica, Jordan, Kazakhstan, Kenya, Kuwait, Kyrgyz Republic, Latvia, Lebanon, Lesotho, Liberia, Lithuania, Luxembourg, Madagascar, Malawi, Maldives, Mali, Malta, Mauritania, Mauritius, Mexico, Moldova, Monaco, Montenegro, Morocco, Mozambique, Namibia, Nepal, Netherlands, Nicaragua, Niger, Nigeria, North Macedonia, Norway, Oman, Pakistan, Panama, Paraguay, Peru, Poland, Portugal, Qatar, Romania, Russian Federation, Rwanda, San Marino, Sao Tome and Principe, Saudi Arabia, Senegal, Serbia, Seychelles, Sierra Leone, St. Vincent and the Grenadines, South Africa, Spain, Sri Lanka, St. Kitts and Nevis, St. Lucia, Sudan, Suriname, Sweden, Switzerland, Syrian Arab Republic, Tajikistan, Tanzania, Togo, Trinidad and Tobago, Tunisia, Turkiye, Turkmenistan, Uganda, Ukraine, United Arab Emirates, United Kingdom, United States, Uruguay, Uzbekistan, Zambia.

**Appendix B: CGI construction**

This study follows the methodology proposed by Sarma [36] to construct the CGI using six WGI’s governance indicators. First, the indicators are categorized under the accountability, transparency, and participation dimensions as follows:

**B1 Accountability dimension**

Two indicators, namely the control of corruption and the rule of law, are assigned to establish the accountability dimension. Control of corruption measures how effectively a country prevents public servants from misusing their positions of power and resources for personal gain. The rule of law measures the extent to which contracts are enforced, property rights are protected, and the likelihood of crime and violence is avoided.

**B2 Transparency dimension**

Under this dimension, regulatory quality and government effectiveness are used as indicators. The government’s effectiveness is determined by the quality of services provided by public firms to citizens that are free from any political influence. It is also the efficacy of the policies that are enacted to strengthen the legitimacy and value of public services. Regulation quality measures how well public capacity is enhanced by government policies to deliver comprehensive and reliable services to the general public and to assist the expansion and development of the private sector.

**B3 Participation dimension**

Political stability and voice and accountability are the relevant indicators for this dimension. Political stability measures how likely it is that a nation will experience upheavals brought on by war, violence, and terrorism. Accordingly, political stability measures the extent to which a state has been negatively impacted by violence and unforeseen circumstances that restrict citizens' freedom to democratically and openly choose and replace leaders. The voice and accountability index assesses a country's capacity to select its own government and exercise its constitutionally guaranteed freedoms of expression, association, and the press.

**B4 Construction process**

The construction of CGI follows a four-step computation. First, using the coefficient of variation $(\vartheta_{i})$, the weight of each indicator is assigned. To that end, the mean value $(\bar{x}_{i})$ and the standard deviation $(\sigma_{i})$ of each indicator is computed. To calculate $(\vartheta_{i})$, the following equation is employed:

$\vartheta_{i}=\sigma_{i}/\bar{x}_{i}, i=1,2,...,=n$ (b1)

and then, using the total sum, the weight of each indicator $(w_{i})$is computed as:

$w_{i}=\vartheta_{i}/\sum_{i=1}^{n} \vartheta_{i},$ (b2)

Table B1 provides complete information about the allocation of wights to each indicator. Second, the normalized value of each indicator is estimated as follows:

$X_{i}=w_{i}\left[\left(A_{t}-L_{i}\right)/\left(U_{i}-L_{i}\right)\right]$ (b3)

Here,$X_{i}$ is the normalized value,$w_{i}$is the assigned weight of the indicator obtained from equation (a2),$A_{i}$is the actual value of the indicator,$U_{i}$is the upper limit set by 90^th^ percentile rank, and $L_{i}$is the lower limit set as zero. The reason for fixing the upper limit, say, at the maximum value of the 90^th^ percentile rank, is based on the maximum value that has been obtained from the descriptive statistics of the governance indicators. Third, the normalized $(X_{1})$and reversed normalized $(X_{2})$values of the dimension are computed as follows:

$\begin{matrix} X_{1}=\sqrt{\left[\left(\sum_{i=1}^{n} X_{i}^{2}\right)/\left(\sum_{i=1}^{n} w_{i}^{2}\right)\right]}, \\ X_{2}=1-\sqrt{\left[\left(\sum_{i=1}^{n} \left(w_{i}-X_{i}\right)^{2}\right)/\left(\sum_{i=1}^{n} w_{i}^{2}\right)\right]}, \end{matrix}$ (b5)

Finally, the CGI can be estimated by taking the average of $(X_{1})$and $(X_{2})$as follows:

$CGI=\frac{X_{1}+X_{2}}{2}$ (b6)

where CGI is the composite governance index used to capture the extensive effects of good governance on the HE and OPHE, augmented as a key variable of interest in the study. The existing literature in finance, economics, and econometrics have widely appreciated the efficiency of this method and preferred over other common techniques used by United Nations Development. CGI is expressed in numbers ranging from 0 to 1.

**Table B1** Allocation of weights-WGI’s indicators.

| Dimension | WGI’s indicators | Allocated weights | | | | | | |
| --- | --- | --- | --- | --- | --- | --- | --- | --- |
|  |  | Full sample |  | High-income |  | Middle-income |  | Low-income |
| Accountability | Control of corruption  Rule of law | 0.75  0.80 |  | 0.75  0.77 |  | 0.40  0.40 |  | 0.19  0.17 |
| Transparency | Regulatory quality  Government effectiveness | 0.75  0.75 |  | 0.77  0.77 |  | 0.38  0.45 |  | 0.17  0.14 |
| Participation | Vice and accountability  Political stability | 0.75  0.70 |  | 0.77  0.67 |  | 0.40  0.40 |  | 0.20  0.20 |
